# Hematological indices in the adult saudi population: Reference intervals by gender, age, and region

**DOI:** 10.3389/fmed.2022.901937

**Published:** 2022-07-28

**Authors:** Naila A. Shaheen, Hina Rehan, Areej Moghairi, Giamal Gmati, Moussab Damlaj, Hind Salama, Mushtaq Rather, May Anne Mendoza, Abeer Alanazi, Bader Al Ahmari, Mohsen Al Zahrani, Ayman Al-Hejazi, Ahmed S. Alaskar

**Affiliations:** ^1^King Saud bin Abdulaziz University for Health Sciences, Riyadh, Saudi Arabia; ^2^Ministry of the National Guard–Health Affairs, Riyadh, Saudi Arabia; ^3^Department of Biostatistics and Bioinformatics, King Abdullah International Medical Research Center, Riyadh, Saudi Arabia; ^4^Divisions of Adult Hematology and SCT, King Abdulaziz Medical City, Riyadh, Saudi Arabia; ^5^King Abdullah International Medical Research Center, Riyadh, Saudi Arabia; ^6^Department of Pathology and Laboratory Medicine, King Abdulaziz Medical City, Ministry of the National Guard–Health Affairs, Riyadh, Saudi Arabia; ^7^Saudi Society of Blood and Marrow Transplant, Riyadh, Saudi Arabia

**Keywords:** complete blood count (CBC), healthy adults, hematological parameters, normal values, reference intervals (RIs)

## Abstract

**Introduction:**

Hematological parameters are critical in disease diagnosis, management, and monitoring; however, complete blood count (CBC) reference intervals vary across populations. The aim of the current study was to provide the reference ranges of hematological parameters/indices in the healthy adult Saudi population.

**Methods:**

A multicenter retrospective cross-sectional study was conducted with a sample of employees who were screened pre-employment from January 2015 to December 2019, at tertiary care hospitals in three regions. Demographic and CBC data were extracted from the electronic health system. The 2.5^th^ and 97.5^th^ percentiles were used to determine the reference intervals.

**Results:**

Of a total of 1,388 participants, 53.82% were male. The majority 96% was less than 40 years old, and 85% were from the Central region. Gender-related differences were observed for the RBC count, Hb, HCT, MCV, MCH, MCHC, and the platelet count. Age-related differences were observed for the RBC, Hb, HCT, and eosinophils. The WBC parameters did not differ by gender or age categories. Region-related differences were observed for the RBC, hemoglobin, HCT, MCV, WBC, and basophils. The platelet count was higher in the female group, the age group 40 years and above, and in the Western region. The prevalence of anemia was high in the female group and the Eastern region. The overall neutropenia rate was 12.8%.

**Conclusion:**

The data from this study provide hematological parameter reference ranges for the adult Saudi population by gender, age, and region. Gender and age-related differences were observed for the hematological parameters. Anemia was more frequent in the female group and the Eastern region. Caution must be taken when comparing or interpreting results from different age groups, gender, region of origin, and ethnicity.

## Introduction

The complete blood count (CBC) is a widely used laboratory test to assess an individual's health and disease status (1). However, several factors can affect the hematological parameters, such as age, gender, ethnicity, lifestyle, environment, and the analytical method of testing. A wide variation has been reported in the hematological parameters of different populations (2, 3). A low normal range has been reported for the hematocrit (HCT), hemoglobin (Hb), and mean platelet volume in the Asian population compared to Caucasian populations (4). Low platelets, white blood cells (WBC), red blood cells (RBCs), and Hb have been reported in South Indian compared to European populations (5). Technical labels of hematological parameters as normal range are not applicable due to the variation in populations. Consequently, normal values or normal range has been replaced by reference values or reference intervals (6). Laboratory Reference Intervals (RIs) are fundamental for disease diagnosis, prognosis, management, or following up the treatment response (7).

The RI values of a laboratory parameter are characterized as 95 ± 2.5% lower and upper limits (between 2.5 and 97.5 percentiles of the result distribution) in a healthy population (8). Laboratories have a best practice of establishing a RI, based on the variables involved. The results are interpreted relative to the normal range of the values of a particular laboratory (9). The Clinical and Laboratory Standards Institute (CLSI) recommends the establishment of RIs for every laboratory (9).

Most of the literature is based on the RIs established in Western populations (10–12). Recent studies reported RIs in the Middle East region (3, 7, 13, 14). A few studies from Saudi Arabia were not comprehensive enough due to a small sample size (15), targeted children and adolescents (16), or focused on one region (17). This study aimed to provide hematological parameter ranges for the adult Saudi population from three regions, and to estimate the prevalence of blood count abnormalities.

## Methods

A multicenter retrospective cross-sectional study was conducted with a sample of adult Saudi employees who were screened during the recruitment process from January 2015 to December 2019, at tertiary care hospitals in three regions (Central, Eastern, and Western). All adult Saudi employees, both male and female, 55 years and younger were included. A positive hepatitis B surface antigen (HBsAg), hepatitis C antibody (anti-HCV), or human immunodeficiency virus antibody (anti-HIV) was an exclusion criterion. The study was approved by the Institutional Review Board, number RC18/094/R. This study was conducted in accordance with the Declaration of Helsinki.

The data were extracted from the electronic health system. The data included age, gender, region, and CBC parameters, such as red blood cells (RBC) count, Hb, HCT, mean corpuscular volume (MCV), mean corpuscular hemoglobin (MCH), mean corpuscular hemoglobin concentration (MCHC), red blood cell distribution width (RDW), white blood cells count (WBC), neutrophils, eosinophils, basophils, lymphocytes, monocytes, and platelets. The initial sample size was 1,438 participants, but 50 records were excluded due to duplicates (*n* = 43), and an age above 55 years (*n* = 7), resulting in a total sample of 1,388.

### Blood sample analysis

Approximately 5.0 ml peripheral venous blood was collected with standard 5 ml K3-EDTA (BD Vacutainer^®^ tubes from Becton, Dickinson, United Kingdom). The blood specimens were processed at the central laboratory of the hospital in each region within 6–8 h of collection. The CBC parameters were measured using ADVIA2120i (Siemens Healthcare Diagnostics, Deerfield, IL, USA) and Cell-Dyn Sapphire (Abbott Laboratories, IL, USA), such as Hb, RBC count, HCT, MCV, MCH, MCHC, RDW, WBC count, WBC differential count (neutrophils, eosinophils, basophils, lymphocytes, and monocytes), and the platelet counts.

### Statistical analysis

The study cohort was divided in three age groups (18–29, 30–39, and ≥ 40 years) for both genders. The effect of gender, age category, and region on the hematological parameters was examined. All the hematological parameters were summarized as mean, and 95% confidence intervals (*CI*). The 95th percentile (2.5 and 97.5 percentiles) with the 95% *CI* was used to determine the reference intervals by gender, age group, and region. The CBC parameters did not follow the Gaussian distribution and the Wilcoxon two-sample test was used to compare the hematological RIs by gender. Kruskal–Wallis test was used to compare the hematological RIs by region and age group.

Blood count abnormalities were defined based on the study hospital's reference intervals: leukopenia if the WBC count was less than 4 (reference range 4.0–11.0 × 10^9^/L), neutropenia if the neutrophil count was <2 (reference range 2.0–7.5 × 10^9^/L), anemia if the Hb was less than 120 (reference range 120–160 g/L), polycythemia (erythrocytosis) if the RBC count was ≥ 5.41 (reference range 4.0–5.40 × 10^12^/L), thrombocytosis if the platelet count was ≥ 401 (reference range 150–400), and thrombocytopenia if the platelet count was <150 (reference range 150–400 × 10^12^/L).

The prevalence of blood count abnormalities was calculated by dividing the frequency of blood count abnormalities by the sample size. The denominator was adjusted by gender and region. The results are reported as % and 95% *CI*. The proportion of blood count abnormalities was compared with a chi-square or Fisher exact test.

All statistical analyses were performed with SAS version 9.4 (SAS Institute Inc., Cary, NC, USA). A *p* < 0.05 was considered statistically significant. The hematological parameters reference intervals were compared with the results reported from different populations within the region and the region as a whole (**Table 6**) (^*^the Hb was compared in g/dl to enable consistent reporting for all the studies).

## Results

**Figure 1:**
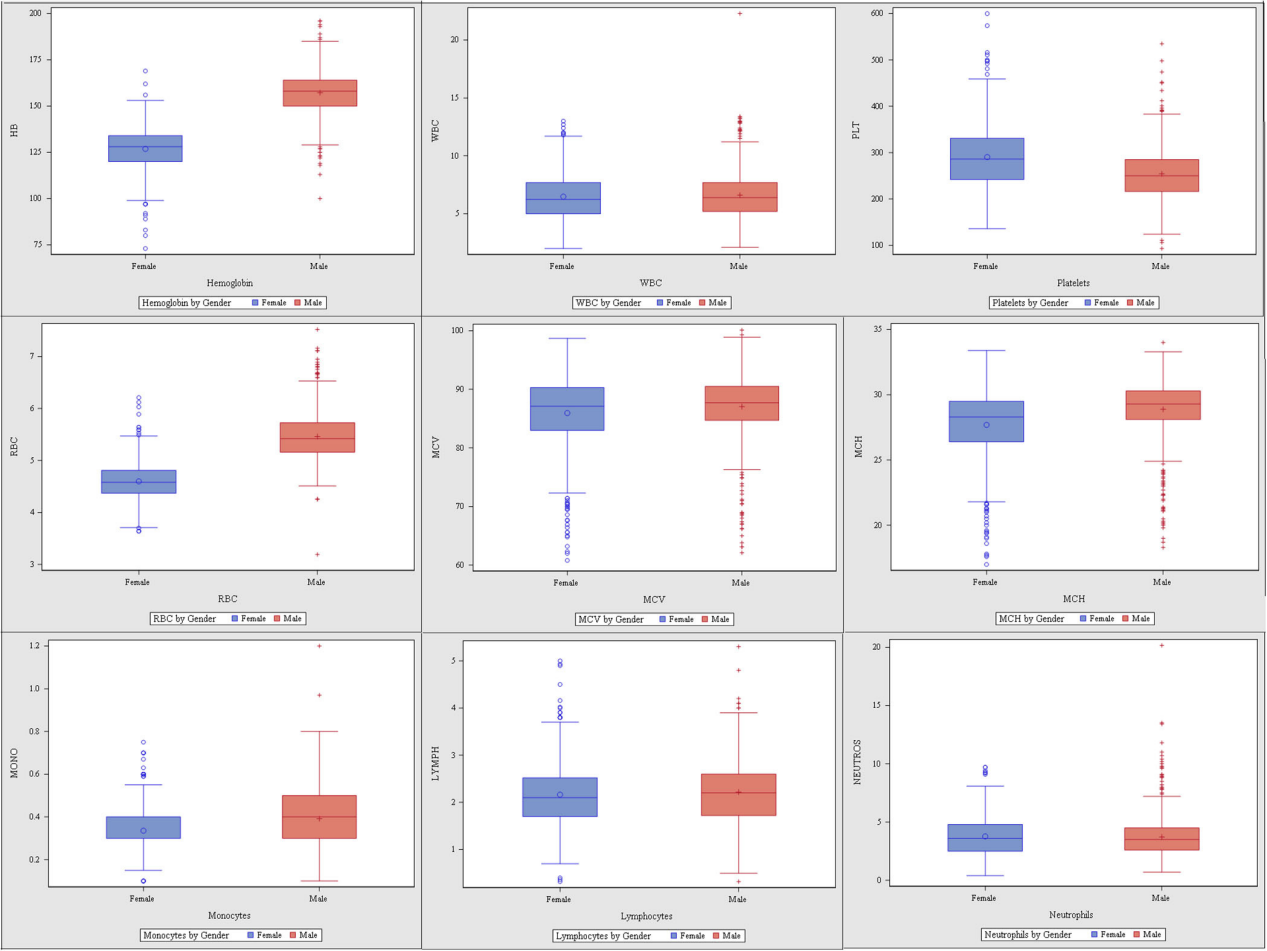
Complete blood count (CBC) parameters ranges of the study cohort by gender.

Of 1,388 participants, 747 (53.82%) were male. The majority (96%, *n* = 1,328) was <40 years (30.61 ± 4.07), and from the Central region (85%, *n* = 1,182) (Supplemental Table 1). The CBC parameters are summarized in Supplemental Table 2 or Figure 1. The CBC reference intervals by gender, age category, and region are summarized in Tables 1–**3**.

**Table 1:** Complete blood count (CBC) reference intervals of the study cohort by gender.

	**Males (*****n** **=*** **747)**				**Females (*****n** **=*** **641)**				
**CBC parameters**	**Mean**	**(95%CI)**	**2.5th percentile**	**97.5th percentile**	**Mean**	**(95%CI)**	**2.5th percentile**	**97.5th percentile**	***p*** **-value**
RDW (%)	13.64	13.57–13.71	12.1	15.9	14.15	14.04–14.26	12.2	17.9	<0.0001
RBC (x 10^12^/L)	5.45	5.42–5.49	4.72	6.59	4.59	4.56–4.62	3.88	5.41	<0.0001
Hemoglobin (g/L)	157.23	156.39–158.0	134	180	126.88	125.97–127.79	101	148	<0.0001
HCT (L/L)	0.53	0.41–0.65	0.42	0.54	0.44	0.34–0.54	0.32	0.45	<0.0001
MCV (fL)	87.05	86.65–87.44	71.2	96	85.94	85.45–86.43	69.7	95.4	0.003
MCH (pg/cell)	28.88	28.73–29.04	22.3	31.9	27.69	27.48–27.89	21	31.4	<0.0001
MCHC (g/L)	331.27	330.12–332.42	305	354	321.28	319.95–322.62	296	345	<0.0001
MPV (fL)	8.52	8.45–8.59	7	10.8	8.48	8.40–8.55	6.8	10.8	0.552
WBC (x 10^9^/L)	6.60	6.45–6.75	3.3	11.2	6.47	6.32–6.63	3.1	11.2	0.335
Neutrophils (x 10^9^/L)	3.71	3.57–3.85	1.2	8.8	3.77	3.63–3.90	1.3	7.6	0.237
Lymphocytes (x 10^9^/L)	2.21	2.16–2.26	1.16	3.6	2.16	2.10–2.21	1.1	3.8	0.102
Monocytes (x 10^9^/L)	0.39	0.38–0.40	0.2	0.7	0.33	0.32–0.34	0.2	0.6	<0.0001
Eosinophils (x 10^9^/L)	2.80	2.65–2.95	0.4	8.1	2.12	2.01–2.25	0.4	6.0	<0.0001
Basophils (x 10^9^/L)	0.70	0.67–0.72	0.2	1.6	0.60	0.57–0.62	0.2	1.3	<0.0001
Platelet (x 10^9^/L)	254.11	250.06–258.17	158	367	290.46	285.16–295.76	178	438	<0.0001

^*^Wilcoxon Rank Sum Test is significant at α ≤ 0.05.

### RBC parameters

Gender-related differences were observed for the RBC count, Hb, HCT, MCV, MCH, and MCHC. The red blood cells in the male group averaged 5.4 (× 10^12^/L), higher than the female group 4.5 (× 10^12^/L). The Hb values in the men averaged 14.9 g/L, higher than the female 12.6 g/L. The MCV and MCHC in men were higher with an average of 87.05 *vs*. 85.94 fL, compared with 331.27 *vs*. 321.28 g/L in women. The mean MCH value in men was 28.9 pg/cell and in women 27.69 pg/cell. The average male-related RDW was 13.64 %, compared with 14.15% in the female group (Table 1). The RBC parameters for the age categories (18–29 years, 30–39 years, 40 years, and above) are summarized in Table 2. The RBC, Hb, and HCT were higher in the 30–39 years age group. Region-related differences were observed for the RBC count, Hb, HCT, MCV, and MPV. The RBC count, Hb, and MCV were higher in the Central region, and the HCT in the Western region. The MPV was higher in the Eastern region (Table 3).

**Table 2:** The CBC reference intervals of the study cohort by age categories.

**CBC parameters**	**Age 18–29 years (*****n** **=*** **765)**				**Age 30–39 years (*****n** **=*** **563)**				**Age 40 and more (*****n** **=*** **60)**				***p*** **-value**
	**Mean**	**(95%CI)**	**2.5th percentile**	**97.5th percentile**	**Mean**	**(95%CI)**	**2.5th percentile**	**97.5th percentile**	**Mean**	**(95%CI)**	**2.5th percentile**	**97.5th percentile**	
RDW (%)	13.91	13.83–13.99	12.2	16.9	13.83	13.72–13.93	12.2	17	13.95	13.66–14.23	12.8	16.1	0.235
RBC (x 10^12^/L)	4.97	4.93–5.02	3.96	6.14	5.17	5.12–5.22	4.12	6.53	5.07	4.94–5.21	4.12	6.12	<0.0001
Hemoglobin (g/L)	141.04	139.67–142.41	104	174	146.47	144.91–148.03	108	180	141.76	137.19–146.33	108	180	<0.0001
HCT (L/L)	0.47	0.38–0.56	0.33	0.52	0.52	0.36–0.68	0.35	0.53	0.43	0.42–0.44	0.35	0.53	<0.0001
MCV (fL)	86.68	86.27–87.09	70.55	95.4	86.40	85.90–86.90	69.5	96	85.96	84.39–87.54	72.3	96.2	0.382
MCH (pg/cell)	28.34	28.17–28.52	21.6	31.7	28.36	28.15–28.57	21.1	31.9	27.93	27.31–28.55	22.6	31.5	0.204
MCHC (g/L)	326.26	325.04–327.49	298	349	327.42	325.94–322.04	300	353	324.81	322.04–327.58	303	345	0.081
MPV (fL)	8.53	8.46–8.61	6.9	10.8	8.47	8.06–8.54	6.9	10.8	8.30	8.06–8.54	6.8	10.8	0.165
WBC (x 10^9^/L)	6.47	6.32–6.61	3.25	10.9	6.63	6.46–6.79	3.2	11.5	6.71	6.12–7.29	3.2	11.4	0.406
Neutrophils (x 10^9^/L)	3.72	3.59–3.86	1.2	7.9	3.74	3.59–3.89	1.3	7.9	3.85	3.32–4.38	1.2	7.6	0.961
Lymphocytes (x 10^9^/L)	2.15	2.10–2.20	1.1	3.6	2.25	2.18–2.31	1.1	3.8	2.15	1.96–2.33	0.8	3.8	0.081
Monocytes (x 10^9^/L)	0.36	0.35–0.37	0.2	0.7	0.37	0.36–0.38	0.2	0.63	0.36	0.32–0.41	0.2	0.7	0.176
Eosinophils (x 10^9^/L)	2.35	2.22–2.48	0.4	7.3	2.65	2.49–2.82	0.4	7	2.79	2.32–3.26	0.7	7.6	0.0001
Basophils (x 10^9^/L)	0.66	0.63–0.68	0.2	1.4	0.64	0.61–0.67	0.2	1.4	0.68	0.58–0.78	0.2	1.5	0.251
Platelet (*x* 10^9^*/L*)	274.51	269.91–279.10	169	420.5	263.43	258.30–268.55	163	392	293.33	272.11–314.56	157	498	0.001

*Kruskal-Wallis Test is significant at α ≤ 0.05.

**Table 3:** The CBC reference intervals of the study cohort by regions.

**CBC parameters**	**Central (*****n** **=*** **1,182)**				**Eastern (*****n** **=*** **130)**				**Western (*****n** **=*** **76)**				
	**Mean**	**(95%CI)**	**2.5th percentile**	**97.5th percentile**	**Mean**	**(95%CI)**	**2.5th percentile**	**97.5th percentile**	**Mean**	**(95%CI)**	**2.5th percentile**	**97.5th percentile**	***p*** **-value**
RDW (%)	13.81	13.74–13.87	12.2	16.8	14.55	14.33–14.76	12.6	17.4	13.83	13.57–14.09	12.1	16.7	<0.0001
RBC (x 10^12^/L)	5.08	5.04–5.11	4.09	6.31	4.89	4.78–5.0	3.74	6.02	5.01	4.88–5.15	3.86	6.41	0.003
Hemoglobin (g/L)	144.3	143.2–145.3	108	177	135.6	132.1–139.0	97	168	140.1	135.7–144.5	99	170	<0.0001
HCT (L/L)	0.44	0.43–0.44	0.35	0.53	0.41	0.40–0.42	0.31	0.50	1.44	0.007–2.88	0.31	33.5	<0.0001
MCV (fL)	86.84	85.52–87.17	71.2	96	84.41	83.23–85.59	66.2	92.5	84.48	83.99–86.97	66.9	96	0.0002
MCH (pg/cell)	28.42	28.28–28.56	21.9	31.8	27.79	27.27–28.31	20	31.7	27.91	27.30–28.51	21.2	31.3	0.039
MCHC (g/L)	327	326.2–327.7	301	350	328.68	326.2–331.1	299	356	318.1	306.9–329.4	34.3	345	0.227
MPV (fL)	8.49	8.43–8.55	6.9	10.8	8.66	8.49–8.84	6.6	10.8	8.34	8.17–8.51	7	10.5	0.022
WBC (x 10^9^/L)	6.58	6.46–6.69	3.4	11.2	6.19	5.78–6.60	2.76	10.1	6.57	6.07–7.07	3.2	11.8	0.034
Neutrophils (x 10^9^/L)	3.73	3.63–3.83	1.2	7.9	4.09	3.18–4.99	1.32	13.51	3.61	3.26–3.96	1.34	6.92	0.889
Lymphocytes (x 10^9^/L)	2.20	2.16–2.24	1.1	3.7	1.79	1.62–1.96	0.58	2.99	2.33	2.14–2.51	1.17	4.16	<0.0001
Monocytes (x 10^9^/L)	0.36	0.35–0.37	0.2	0.7	0.35	0.30–0.39	0.2	0.7	0.37	0.34–0.40	0.15	0.67	0.409
Eosinophils (x 10^9^/L)	2.49	2.39–2.60	0.4	7.2	2.921	2.15–3.68	0.4	8.5	2.211	1.88–2.54	0.5	6	0.064
Basophils (x 10^9^/L)	0.65	0.63–0.67	0.2	1.4	0.78	0.62–0.94	0.2	2.2	0.66	0.58–0.73	0	1.7	<0.0001
Platelet (*x* 10^9^*/L*)	271.04	267.3–274.7	163	418	260.5	249.5–271.5	161	416	285.7	271.6–299.8	193	492	0.016

*Kruskal-Wallis Test is significant at α ≤ 0.05.

### WBC parameters

Gender-related differences were not observed in the WBC, neutrophils, and lymphocytes. However, gender-related differences were observed in the male compared to female, for the monocytes (0.39 *vs*. 0.33 × 10^9^/L), eosinophils (2.80 *vs*. 2.12 × 10^9^/L), and basophils (0.70 *vs*. 0.60 × 10^9^/L) (Table 1). The WBC parameters did not differ by age category except for the eosinophils (Table 2). Region-related differences were observed for the WBC, lymphocytes, and basophils (Table 3).

### Platelets parameters

The gender-related difference was observed for the platelet count, which was higher in female (290.46 *vs*. 254.1 × 10^9^/L) (Table 1). The platelet count varied by age category and was higher in the 40 year and older age group (Table 2). The platelet count was higher in the Western region (Table 3).

### The prevalence rate of leukopenia, neutropenia, anemia, polycythemia, thrombocytosis, and thrombocytopenia by gender and region

The most frequent blood abnormalities identified in the study cohort are summarized in Tables 4, 5. Gender-related differences were observed in the rate of anemia, polycythemia, thrombocytosis, and thrombocytopenia. The overall rate of anemia was 10.6% (95% *CI*: 9.15, 12.42), and higher in women 22.5% (95% *CI*: 19.45, 25.96). The rate of polycythemia was higher in men 51.6% (95% *CI*: 48.02, 55.22). Region-related differences were observed in the rate of anemia, and neutropenia which was noticeably higher in the Eastern region 22.48% (95% *CI*: 16.13, 30.42), and Central region 12.97% (95% *CI*: 11.13, 15.07) (Table 5).

**Table 4:** Prevalence rate of leukopenia, neutropenia, anemia, polycythemia, thrombocytosis, and thrombocytopenia by gender.

	**Overall** ***n** **=** **1388***	**Males** ***n** **=** **747***	**Females** ***n** **=*** **641**	***p-value***
	**Rate % (95%CI)**	**Rate % (95%CI)**	**Rate % (95%CI)**	
Leukopenia	7.01 (5.78–8.49)	6.23 (4.71–8.21)	7.92 (6.06–10.3)	0.220^*^
Neutropenia	12.82 (11.08–14.80)	13.35 (10.99–16.13)	12.19 (9.75–15.14)	0.542^*^
Anemia	10.67 (9.15–12.42)	0.54 (0.21–1.39)	22.54 (19.45–25.96)	<0.0001^*^
Polycythemia	28.97 (26.63–31.43)	51.63 (48.02–55.22)	2.54 (1.57–4.08)	<0.0001^*^
Thrombocytosis	3.51 (2.65–4.62)	1.08 (0.55–2.12)	6.34 (4.69–8.52)	<0.0001^*^
Thrombocytopenia	0.66 (0.35–1.24)	1.08 (0.55–2.12)	0.16 (0.03–0.89)	0.043^**^

* *Chi-square test*.

** *Fisher exact test*.

*The table is based on the current reference intervals used in the study hospital*.

**Table 5:** Prevalence rate of leukopenia, neutropenia, anemia, polycythemia, thrombocytosis, and thrombocytopenia by region.

	**Overall** ***n** **=** **1,388***	**Central** ***n** **=** **1,182***	**Eastern** ***n** **=** **130***	**Western** ***n** **=** **76***	***p-value***
	**Rate % (95%CI)**	**Rate % (95%CI)**	**Rate % (95%CI)**	**Rate % (95%CI)**	
Leukopenia	7.01 (5.78–8.49)	6.87 (5.56–8.47)	10.08 (5.98–16.48)	3.95 (1.35–10.97)	0.224^**^
Neutropenia	12.82 (11.08–14.80)	12.97 (11.13–15.07)	12.50 (5.86–24.70)	10.81 (5.58–19.91)	0.863^*^
Anemia	10.67 (9.15–12.42)	9.19 (7.66–10.99)	22.48 (16.13–30.42)	13.33 (7.41–22.83)	<0.0001^*^
Polycythemia	28.97 (26.63–31.43)	30.12 (27.55–32.82)	20.16 (14.14–27.89)	26.32 (17.73–37.18)	0.052^*^
Thrombocytosis	3.51 (2.65–4.62)	3.61 (2.68–4.84)	3.10 (1.20–7.70)	2.63 (0.72–9.1)	1.000^**^
Thrombocytopenia	0.66 (0.35–1.24)	0.77 (0.41–1.46)	0	0	-

* *Chi-square test*.

** *Fisher exact test*.

*The table is based on the current reference intervals used in the study hospital*.

### Comparison of reference intervals globally

Table 6 summarizes the hematological parameter RIs in different populations in the region and other populations. The MCV in men and women is lower in Saudi Arabia compared to Caucasians (France and Canada), but similar to Nigeria. However, it is lowest in Mali and Iran. The lower limit of Hb is higher in Saudi men compared to Omani men, but similar to the Kuwaiti population. The lower limit of Hb in Saudi women is lower than France, Canada, Mali, Kenya, Oman, and Kuwait. The lower limit of neutrophils is lower in Saudi Arabia compared to Caucasians; however, it is lowest in Brazil and Oman.

**Table 6:** Comparison of CBC parameters reference intervals across reported studies.

**CBC parameters**		**Saudi Arabia 2022**	**France 2014** [1]	**Canada 2015** [35]	**Kuwait 2016** [14]	**Mali 2017** [2]	**Oman 2018** [3]	**Kenya 2018** [41]	**Korea 2018** [27]	**Thai 2018** [23]	**Nigeria 2019** [25]	**Brazil 2019** [10]	**Iraq 2020** [13]	**Iran 2021** [7]
	Age ^*^	18–55	20–59	20–79	18–96	18–59	18–69	20–65	15–99	18–60	18–26	18–59	18–39	35–65
RBC (x10^12^/L)	*M*	4.72–6.59	4.45–5.72	4.3–5.7	4.7–5.8	4.15–6.24	4.45–6.75	4.94–6.52	4.1–5.6	4.2–6.1	4.4–7.0	4.4–5.8	–	4.32–6.01
	*F*	3.88–5.41	3.97–5.14	3.8–5.0	4.1–5.5	3.88–5.75	4.07–6.17	4.31–5.76	3.8–5.1	4.0–5.5	4.4–6.8	3.9–5.1	–	4.06–5.62
Hemoglobin (g/dL)	*M*	13.4–18.0	13.4–16.7	13.6–16.9	13.8–16.4	12.4–17.5	12.4–16.4	14.5–18.7	13.0–17.0	12.7–16.9	12.0–17.2	13.0–16.9	13.6–17.4	12.3–16.8
	*F*	10.1–14.8	11.5–14.9	11.9–14.8	11.3–13.9	12.0–14.9	11–15.1	12.0–16.5	11.1–14.8	12–14.9	11.7–17.0	11.5–14.8	11.9–15.5	11.2–15.4
HCT (L/L)	*M*	0.42–0.54	0.39–0.48	0.40–0.50	0.41–0.50	0.33–0.54	0.36–0.47	0.43–0.55	0.38–0.5	0.40–0.51	–	0.39–0.52	0.39–0.51	0.35–0.47
	*F*	0.32–0.45	0.34–0.43	0.35–0.43	0.34–0.42	0.26–0.52	0.33–0.43	0.36–0.49	0.33–0.44	0.37–0.45	–	0.35–0.46	0.34–0.45	0.32–0.42
Platelet (x *10^9^/*L)	*M*	158–367	172–398	151–324	184–304	142–482	146–347	133–356	159–367	160–356	89–374	128–302	139–339	161–337
	*F*	178–438	185–445	153–361	204–350	151–532	164–368	152–443	159–367	179–435	101–425	137–344	158–405	174–363
MCV (fL)	*M*	71.2–96.0	79.5–93.6	82.5–98	80–93	72.1–96.4	62.5–88.5	76.5–95.5	83.3–98.0	80.6–98.8	70.0–96.0	81.5–100.2	–	65.3–90.1
	*F*	69.7–95.4	76.1–94.0	82.5–98	77–92	39.2–118	62.5–88.5	73.4–95.8	83.3–98.0	80.4–95.9	69.2–95.0	81.0–100.1	–	64.1–89.6
MCH (pg/cell)	*M*	22.3–31.9	27.3–32.8	27.6–33.3	26–32	23.1–32.9	20.81–31.2	25.1–32.8	27.7–33.2	25.8–33.1	21.3–31.5	26.9–32.5	–	22.1–32.9
	*F*	21–31.4	26.4–32.6	27.6–33.3	25–31	23.1–34.8	20.81–31.2	24.4–32.7	27.7–33.2	25.0–31.2	21.0–31.2	26.3–32.3	–	21.7–32.9
MCHC (g/L)	*M*	30.5–35.4	32.4–36.3	32.5–35.2	32.7–34.7	30.9–34.5	31–37.2	32.4–35.4	323–359	30.8–34.6	29.0–35.0	30.6–34.6	–	32.9–37.8
	*F*	29.6–34.5	31.9–35.8	32.5–35.2	32.3–34.1	30.9–34.5	31–37.2	32.0–35.0	317–351	30.2–34.2	28.0–34.8	30.5–34.3	–	32.8–37.7
WBC (x10^9^/L)	*M*	3.3–11.2	4.09–11	3.8–10.4	5.9–12	2.97–8.80	2.79–8.09	3.13–8.10	4.0–10.3	4.5–11.3	3.4–9.6	2.8–9.4	4.4–10.6	4.1–9.3
	*F*	3.1–11.2	4.02–11.42	3.8–10.4	5.4–11	3.8–12.5	2.79–8.09	2.89–7.72	3.5–9.6	4.4–10.3	3.6–10.3	2.9–10.04	4.6–11	4.1–9.3
Neutrophils (x 10^9^/L)	*M*	1.2–8.8	1.78–6.94	1.8–7.2	2.7–8.0	1.0–4.4	0.91–4.61	1.02–3.92	1.48–7.21	1.8–7.9	1.2–5.6	0.55–5.9	–	–
	*F*	1.3–7.6	1.75–7.50	2.0–7.4	2.6–8.0	1.2–7.4	0.91–4.61	1.07–4.42	1.13–6.15	1.76–7.51	1.3–6.0	0.59–6.55	–	–
Lymphocytes (x 10^9^/L)	*M*	1.16–3.6	1.34–3.91	1.0–3.2	1.5–3.0	1.2–3.8	1.12–3.15	1.36–3.58	–	1.3–3.5	1.1–4.3	0.76–3.40	–	1.09–5.18
	*F*	1.1–3.8	1.37–3.96	1.0–3.2	1.4–3.0	1.4–4.6	1.12–3.15	1.22–3.24	–	1.2–3.4	1.2–4.3	0.82–3.49	–	1.08–5.18
Monocytes (x 10^9^/L)	*M*	0.2–0.7	0.22–0.77	0.2–0.8	0.4–1.0	0.1–0.67	0.23–0.72	0.15–0.76	0.03–0.10	0.2–0.9	–	0.01–0.81	–	0.07–0.53
	*F*	0.2–0.6	0.20–0.71	0.2–0.8	0.4–1.0	0.1–0.5	0.23–0.72	0.14–0.68	0.03–0.10	0.2–0.6	–	0.19–0.68	–	0.07–0.53
Eosinophils (x 10^9^/L)	*M*	0.4–8.1	0.04–0.57	0.1–0.2	<0.01–0.5	0.0–0.1	0.03–0.37	0.05–0.64	0.004–0.07	0.8–9.2	–	0.0–0.64	–	–
	*F*	0.4–6.0	0.04–0.51	0.1–0.2	<0.01–0.4	0.0–0.82	0.03–0.37	0.04–0.49	0.004–0.07	0.4–7.5	–	0.0–0.54	–	–
Basophils (x 10^9^/L)	*M*	0.2–1.6	0.0–0.09	0.0–0.1	0.01–0.1	0.0–0.11	0.002–0.05	0.01–0.08	0.001–0.01	0.0–0.1	–	0.0–0.62	–	–
	*F*	0.2–1.3	0.04–0.51	0.0–0.1	0.01–0.1	0.0–0.10	0.002–0.05	0.01–0.06	0.001–0.01	0.01–0.09	–	0.0–0.73	–	–
RDW	*M*	12.1–15.9	–	11.4–13.5	12.5–15	11.9–17.4	11.1–17.8	11.3–14.7	11.9–14.3	11.9–14.5	–	12.0–15.2	–	9.9–12.7
	*F*	12.2–17.9	–	11.4–13.5	12.4–16	11.6–24.3	11.1–17.8	11.4–15.8	11.9–14.3	11.7–15.0	–	11.9–15.4	–	9.9–12.7

* *Complete Blood Count (CBC), included Age (years) range in each study*.

*The Hemoglobin is reported as g/dL only for this table for comparison across studies*.

*The reported values in percentages are converted to 10^9^/L*.

## Discussion

This study provided the RIs for the adult Saudi population for three age categories, gender, and regions. The establishment of hematological RIs in terms of disease diagnosis and monitoring is crucial. Hematological parameters are influenced by age, gender, and lifestyle (8). Ethnicity is also a factor to consider when establishing RIs for hematological parameters (18). Canada endorsed specific RIs established by the Canadian Laboratory Initiative for Pediatric Reference Intervals (CALIPER), (19) and the Canadian Health Measures Survey (CHMS) (20). Australia also reported specific RIs for the population (21). In the last decade, several studies reported RIs form different parts of the world (2, 7, 10, 13, 22–29), such as Saudi Arabia (15–17). The reported hematological parameters RIs in literature differ for populations, for example, Asian Americans and American blacks compared to white Americans (4), South Indians compared to European (5) and Africans compared to Western populations (30, 31). Few studies recently reported hematological RIs in the Middle Eastern region (3, 7, 13, 14).

In the current study, compared to men, women had lower RBC, Hb, HCT, MCV, and MCHC. The findings that men have higher levels of RBC, Hb, HCT, MCV, and MCHC parameters are consistent as reported in literature (7, 10, 27–29, 32, 33). The findings are also consistent with a regional study (3). The gender-related differences in the RBC parameters were noticeable after the age of 14 years (27). A possible explanation for the gender difference in Hb is low iron stores due to menstruation, decreased muscle mass, the effect of androgens/estrogen, and decreased metabolic demand (3, 34, 35). A Canadian study also reported changes in hemoglobin by gender overtime (35).

In the current study, the lower limit of Hb in the female group was lower compared with the Caucasians (France and Canada) (1, 35) and also the populations in Oman and Kuwait (3, 14). A possible explanation of the Hb difference compared to Caucasians is the prolonged menstrual cycle in Saudi women [median bleeding days were 5 (1–16)] (36). Other reasons could be lifestyle, dietary habits, and due to the prevalence of other blood disorders (37, 38).

A study from the Aljouf region, Saudi Arabia (17) reported that the RIs differed from other studies reported in the region (Tanzania and Palestine) (39, 40). An interesting observation was that the RIs reported in the Aljouf region differed slightly from the current study (17) which warranted further exploration and indicated that RIs could be different not only for populations but also between regions of a country. Possible reasons are changes in altitude, climate, and lifestyle. Interestingly, the RIs of the current study differ from other populations in the region, however, with similarities to the Kenyan and Thai population (23, 41).

Gender-related differences were not observed for the WBC, neutrophils, and lymphocytes in the current study, which contradicts the previous findings (33). The current study indicates significant difference in the RDW by gender, also contrasting a prior study (10). Genetics play a key role in the platelet count variation in populations (42). In general, a lower platelet count has been reported in men compared to women, probably due to hormones or compensation due to menstruation (34). In the current study, the platelet-related findings are consistent with literature (1, 27, 32, 42). A possible explanation for the higher platelet count in women could be due to estrogen-promoting platelet production (43). Another factor related to higher platelets is a reduction in the body iron stores. A moderate iron deficiency stimulates the platelet production due to iron deficiency (44, 45). However, a study also reported no gender effect on the platelet count (28).

The comparison of an age-related variation is not possible as the reported age group is not homogeneous in all the studies (10, 27). A comprehensive Korean study included all age groups, namely pediatric, adults, and geriatric (27). The age groups of the current study are similar to the Kenyan study (41). The age-specific CBC parameters trends observed in the current study were similar to an another study (35). The RBC, Hb, and HCT were higher in the 30–39 years age group. As reported in literature, age influences the Hb, the value gets lower with increasing age (46). The RDW increases with age as reported earlier (47, 48), which contradicts the findings of the current study with no significant difference in RDW between the age categories. The mean platelet count in the current study decreased with advancing age, supporting literature (7, 42). A possible rationale for the reduction in the platelet count is the decrease in the hematopoietic stem cell reserve with aging (42). However, no difference in an age-related variation has also been reported (41).

In the current study, the prevalence of blood abnormalities is based on the study hospital reference ranges. The cutoff for anemia, as described by the World Health Organization (WHO) is based on the severity of the anemia (49). We have observed a lower rate of anemia (10.6%), compared to Oman (28%) (50). In the current study, based on the WHO guidelines, anemia occurred more frequently in women. As reported by WHO 2011 report, the prevalence of anemia in women (22.5%) is lower compared to the child bearing age of women globally (29.4%) (51). Several studies reported a prevalence of anemia in Saudi Arabia, from the Eastern province 35.5% (71/201) (52), 67.35% (33/49) from Jazan (53), and 64% (171/268) from Madinah (54). The reported prevalence of sickle cell disease is highest in the Eastern region (54) and ranges between 2 and 27% (55, 56), which explains the higher rate of anemia in the Eastern region. The current study results indicate the prevalence of polycythemia/erthyrocytosis (29%), which is lower than a prior Saudi study (24/200, 38.7%) (57). The polycythemia is higher among men the fact which can be attributed to the smoking status among men. However, the smoking status for all participants could not be captured due to the retrospective nature of the data. Benign neutropenia is a frequent finding in our clinical practice, also reported in literature (58–60). One study reported a prevalence of 20%, but the sample size was only 69 (60). The overall prevalence rate of benign neutropenia (12.8%) in the current study falls within the range reported by the Western region of Saudi Arabia (11–23%) (58). However, in the current study, the benign neutropenia rate is higher in the Central region (12.97%) *vs*. the Western region (10.8%). Our benign neutropenia rate is slightly higher than reported by studies done in the region, such as Qatar (10.7%) (61) and the United Arab Emirates (10.7%) (62). However, the cut-off for defining neutropenia in the study from Qatar was lower (1.5 × 10^9^/L) than the current study (61). The key point is that benign neutropenia is frequently reported in Arabs (60, 62), possibly due to genetic factors (63). In addition, an interesting discussion explores the role of altitude in the neutrophil count (59). In the current study, the neutropenia rate is higher than reported in the West, such as Black (4.5%) and White Americans (0.79%) (64). A strong association was reported between being from an African origin and neutropenia, ranging from 2.7 to 12.5% (65).

### Limitations

The study has several limitations. Firstly, the results may not represent the hematological reference range for Saudi Arabia, and are not applicable to children, adolescents, and the elderly. Secondly, the employees from different regions are a mixed group, for example, participants from a region residing in another region and not mutually exclusive. Due to the retrospective nature of the data, it was not possible to screen for chronic medical conditions (diabetes mellitus or hematological diseases). Thirdly, the information related to the factors which might influence the reference intervals, such as inflammation-related disease, drugs, occupational exposure, circadian rhythms, and the blood sample collection time could not be gathered.

## Conclusion

The data from this study provide hematological parameter reference ranges for a healthy adult Saudi population by gender, age, and region. Gender- and age-related differences were observed for the hematological parameters. Anemia was more frequent in the female and the Eastern region. The rate of benign neutropenia was similar to a study reported in Saudi Arabia; however, it was higher than studies reported in the region. Our findings also reflect that some hematological reference intervals were not only different from Western populations, but also from some of the other populations in the region. Caution must be taken when comparing or interpreting results from the different age groups, gender, regions of origin, and ethnicity. We recommend additional large-scale studies exploring all the influencing factors and age groups prior to establishing reference intervals.

## Data availability statement

The datasets generated and/or analyzed during the current study are not publicly available due to the institutional rules and regulations but are available from the author on a reasonable request. Requests to access these datasets should be directed to askaras@ngha.med.sa.

## Ethics statement

The studies involving human participants were reviewed and approved by King Abdullah International Medical Research Center Institutional Review Board. Written informed consent for participation was not required as secondary data was used and there was no direct interaction with the participants.

## Author contributions

All authors made significant contributions to the conception, study design/execution of the study, data acquisition, analysis, interpretation, drafting manuscript, and critical review. The final version of the manuscript has been reviewed by all the authors and all agreed on the journal chosen for publication.

## Conflict of interest

The authors declare that the research was conducted in the absence of any commercial or financial relationships that could be construed as a potential conflict of interest.

## Publisher's note

All claims expressed in this article are solely those of the authors and do not necessarily represent those of their affiliated organizations, or those of the publisher, the editors and the reviewers. Any product that may be evaluated in this article, or claim that may be made by its manufacturer, is not guaranteed or endorsed by the publisher.
