# Supplementary material for: Hematological indices in the adult saudi population: Reference intervals by gender, age, and region
Source: Front Med (Lausanne). 2022 Jul 28;9:901937. doi: 10.3389/fmed.2022.901937 (PMC9366111; doi:10.3389/fmed.2022.901937)
Supplement: Supplementary file 1 [file Table_1.DOCX]

**Supplemental Table 1: Demographic Characteristics of the Study Cohort**

| **Characteristics** |  |
| --- | --- |
| **Gender** *n(%)* n=1388 |  |
| *Male* | 747(53.82) |
| *Female* | 641(46.18) |
| **Regions** *n(%)*n=1388 |  |
| *Central* | 1182(85.16) |
| *Eastern* | 130(9.37) |
| *Western* | 76(5.48) |
| **Age** *(mean±SD),* *range* | 30.61± 4.07, (25–54) |
| **Age categories** *n(%)* n=1388 |  |
| *18–29 years* | 765(55.12) |
| *30–39 years* | 563(40.56) |
| *≥ 40 years* | 60(4.32) |
| **Smoking** n=1255 |  |
| *Yes* | 201(16.02) |
| *No* | 1255(83.98) |
| Males *n=747 (smoking status yes)* | 186(24.89) |
| Females *n=641 (smoking status yes)* | 15/641(2.34) |
