# Supplementary material for: Hematological indices in the adult saudi population: Reference intervals by gender, age, and region
Source: Front Med (Lausanne). 2022 Jul 28;9:901937. doi: 10.3389/fmed.2022.901937 (PMC9366111; doi:10.3389/fmed.2022.901937)
Supplement: Supplementary file 2 [file Table_2.DOCX]

**Supplemental Table 2: CBC Parameters Ranges of the Study Cohort**

| **CBC Parameters** | **Males**  n=747  *(mean±SD)* | **Females**  n=641  *(mean±SD)* |
| --- | --- | --- |
| **RDW** (%) [11.5–14.5]* n=1369 | 13.65±0.94 | 14.16±1.35 |
| **RBC** (x 10^12^/L) [4.0–5.40] n=1367 | 5.46±0.46 | 4.60±0.38 |
| **Hemoglobin**(g/L) [120–160] n=1368 | 157.24±11.62 | 126.88±11.66 |
| **HCT** (L/L) [0.36–0.54] n=1369 | 0.53±1.63 | 0.45±1.32 |
| **MCV** (fL) [76.0–96.0]n=1369 | 87.05±5.44 | 85.95±6.29 |
| **MCH** (pg/cell) [27.0–32.0] n=1369 | 28.89±2.19 | 28.89±2.19 |
| **MCHC** (g/L) [320–350] n=1369 | 331.28±15.88 | 321.29±17.11 |
| **MPV** (fL) [6.3–10.3] n=1369 | 8.53±1.03 | 8.48±1.01 |
| **WBC** (x 10^9^/L) [4.0–11.0] n=1369 | 6.60±2.06 | 6.48±1.98 |
| **Neutrophils** (x 10^9^/L) [2.0–7.5] n=1240 | 3.72±1.86 | 3.77±1.64 |
| **Lymphocytes** (x 10^9^/L) [2.0–7.50] n=1240 | 2.22±0.66 | 2.16±0.67 |
| **Monocytes** (x 10^9^/L) [0.10–1.10] n=1240 | 0.39±0.14 | 0.34±0.11 |
| **Eosinophils** (x 10^9^/L) [0.10–0.70] n=1238 | 0.18±0.13 | 0.14±0.11 |
| **Basophils** (x 10^9^/L) [0.0–0.10] n=1240 | 0.04±0.05 | 0.02±0.04 |
| **Platelet** (x 10^9^*/*L) n=1369 | 254.12±56.08 | 290.47±67.83 |

**Complete blood count reference ranges of the study hospital*
